# High-Throughput Plant Phenotyping Platform (HT3P) as a Novel Tool for Estimating Agronomic Traits From the Lab to the Field

**DOI:** 10.3389/fbioe.2020.623705

**Published:** 2021-01-13

**Authors:** Daoliang Li, Chaoqun Quan, Zhaoyang Song, Xiang Li, Guanghui Yu, Cheng Li, Akhter Muhammad

**Affiliations:** ^1^National Innovation Center for Digital Fishery, China Agricultural University, Beijing, China; ^2^Beijing Engineering and Technology Research Centre for Internet of Things in Agriculture, China Agricultural University, Beijing, China; ^3^China-EU Center for Information and Communication Technologies in Agriculture, China Agriculture University, Beijing, China; ^4^Key Laboratory of Agriculture Information Acquisition Technology, Ministry of Agriculture, China Agricultural University, Beijing, China; ^5^College of Information and Electrical Engineering, China Agricultural University, Beijing, China; ^6^Department of Psychology, College of Education, Hubei University, Wuhan, China

**Keywords:** crop improvement, high-throughput, phenomics, phenotyping platform, plant science, remote sensing, sensors

## Abstract

Food scarcity, population growth, and global climate change have propelled crop yield growth driven by high-throughput phenotyping into the era of big data. However, access to large-scale phenotypic data has now become a critical barrier that phenomics urgently must overcome. Fortunately, the high-throughput plant phenotyping platform (HT3P), employing advanced sensors and data collection systems, can take full advantage of non-destructive and high-throughput methods to monitor, quantify, and evaluate specific phenotypes for large-scale agricultural experiments, and it can effectively perform phenotypic tasks that traditional phenotyping could not do. In this way, HT3Ps are novel and powerful tools, for which various commercial, customized, and even self-developed ones have been recently introduced in rising numbers. Here, we review these HT3Ps in nearly 7 years from greenhouses and growth chambers to the field, and from ground-based proximal phenotyping to aerial large-scale remote sensing. Platform configurations, novelties, operating modes, current developments, as well the strengths and weaknesses of diverse types of HT3Ps are thoroughly and clearly described. Then, miscellaneous combinations of HT3Ps for comparative validation and comprehensive analysis are systematically present, for the first time. Finally, we consider current phenotypic challenges and provide fresh perspectives on future development trends of HT3Ps. This review aims to provide ideas, thoughts, and insights for the optimal selection, exploitation, and utilization of HT3Ps, and thereby pave the way to break through current phenotyping bottlenecks in botany.

## Introduction

The growth and development of plants, involving their photosynthesis, transpiration, flowering, and fruiting processes, are the basis of life on earth, and support 7.5 billion people (Pieruschka and Schurr, 2019). Unfortunately, the agriculture that sustains humanity is now facing three stark challenges at once: climate change, resource depletion, and population growth (Kim, 2020). In the next 30 years, the global population is expected to grow by 25% to 10 billion (Hickey et al., 2019). One of the greatest challenges in the twenty-first century will be to quickly expand crop production to meet this growing demand for food, clothing, and fuel. Salinization and erosion of agricultural land around the world, coupled to declining phosphate reserves, pose a grave threat to growth in the global production of crops. On April 21, 2020, the World Food Programme (WFP) announced that as new coronavirus pandemic spreads and batters the global economy, the number of people facing severe food crisis in the world could increase to 265 million within the year. In the past decade, cheaper and faster sequencing methods have fostered increasing crop yields and generated an enormous increase in plant genomic data. The costs of sequencing have fallen dramatically, from $0.52 per Mb of DNA sequence in 2010 to just $0.010 in 2019, while the cost of sequencing a human-sized genome has decreased from $46 774 to a relatively paltry $942 (National Human Research Institute). Although high-throughput genotyping is expanding exponentially, the collection and processing of plant phenotypes constrain our ability to analyze the genetics of quantitative traits and limit the use of breeding for crop yield improvement (Mccouch et al., 2013).

The phenotype arises from interactions between genotype and environment (Hickey et al., 2019), and the essence is the temporal expression of the plants' gene map in characteristic geographic regions (Zhao, 2019). Phenotyping applies specific methods and protocols to measure morphological structural traits, physiological functional traits, and component content traits of cells, tissues, organs, canopy, whole plants, or even populations. However, traditional breeders perform artificial phenotyping based on the appearance, taste, and touch of the crop, undoubtedly a time-consuming, labor-intensive, and even destructive method that requires immense human resources to sample large population of crop plants. The limitation of phenotyping efficiency is increasingly recognized as a key constraint of progress in applied genetics, especially the time interval for acquiring traits in different environments (Guzman et al., 2015). Further, conventional phenotyping methods also make it difficult to capture physiological and biochemical phenotypes at the level of plant basic mechanisms that reveal patterns of genetics and biology. So, to alleviate this bottleneck, since 2000 a variety of phenotyping platforms have been developed which are now common tools in commercial or research teams (Granier and Vile, 2014).

**Figure 1:**
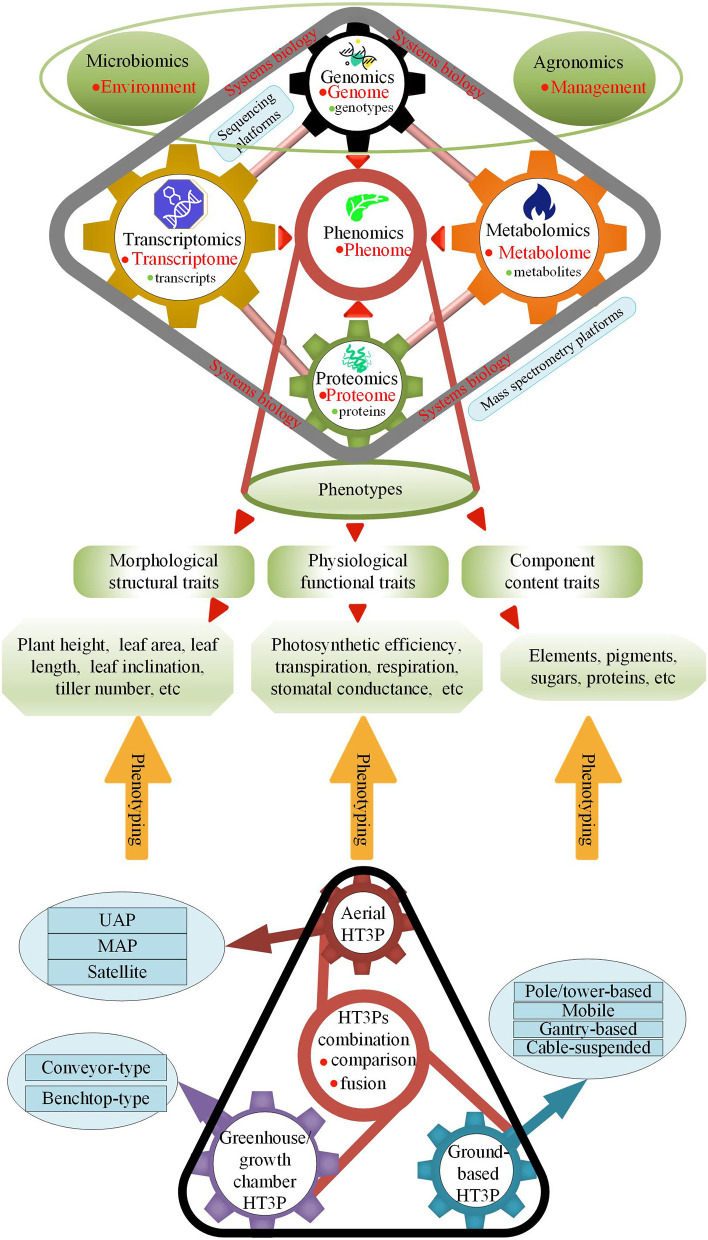
HT3Ps employed for phenotyping plant phenotypes of genotype, environment, and management (G×E×M) interactions advance phenomics; sequencing platforms employed for researching genotypes and transcripts assist in genomics and transcriptomics; mass spectrometry platforms employed for researching proteins, and metabolites promote proteomics and metabolomics; -omics platforms further progress multi-omics in systems biology.

An image-based, high-throughput phenotyping platform (HT3P) is defined as a platform that can image at least hundreds of plants daily (Fahlgren et al., 2015b). Given that some HT3Ps currently not only rely on images but also are based on contact (albeit non-destructive), “HT3P” is defined here as a platform that can collect massive amounts of phenotypic data from hundreds of plants every day with a high degree of automation. HT3P is a novel and powerful tool allowing us to monitor and quantify crop growth and production-related phenotypic traits in a non-destructive, fast, and high-throughput manner, and then to achieve genomics-assisted breeding (GAB) through genomic approaches of quantitative trait loci (QTL) mapping, marker assisted selection (MAS), genomic selection (GS), and genome-wide association studies (GWAS), thereby assisting crop growers to adapt to changing climate conditions and market demand for yield. When genomics and high-throughput phenotypic data are robustly linked together, this fusion will also greatly promote the development of phenotyping. Furthermore, as Figure 1 shows, various types of HT3Ps contribute to the phenotyping of plant morphological structure, physiological function, and fractional content, and they can further promote the developments of multi-omics and reveal the regulatory networks and biological patterns of plants' growth and development.

Nevertheless, because the large phenotyping platforms mostly are developed by professional commercial companies, the underlying hardware and software are protected by patents, so they cannot be modified to meet specific research needs (Czedik-Eysenberg et al., 2018). Consequently, a diversified range of commercial phenotyping platforms, as well as those either customized or self-developed, are continuously emerging. In this context, this paper reviews HT3Ps (root phenotyping not included) under three scenarios: (1) greenhouses and growth chambers under strictly controlled conditions; (2) ground-based proximal phenotyping in the field, and; (3) aerial, large-scale remote sensing, with an emphasis on platform novelties, sensor configurations, operation modes, and applications. Then, we innovatively propose ways to combine HT3Ps for their comparative validation or comprehensive analysis. Finally, we discuss some prevailing issues in current high-throughput phenotyping and also highlight the prospects for future development of HT3P. We hope this review enables researchers on plant phenotyping to make more informed choices when employing HT3P, provides fresh ideas and thoughts for intrepid developers of HT3P, and that ultimately hastens the next green revolution in crop breeding.

## HT3P For Indoor Phenotyping Under Strictly Controlled Environmental Conditions

High-throughput plant phenotyping in the growth chamber or greenhouse entails the precise control of environmental factors—temperature, humidity, gas concentration, air volume, wind speed, light intensity, spectral range, photoperiod, and nutrient content—and a high-throughput, non-destructive, highly repeatable, fast, and accurate capture of the plant response to a specific environment. This can be done using model crops or representative plants as research objects, and the analysis of plants' structure, physiology, and biochemical characteristics with assistance of HT3P can reveal adaptive mechanisms related to environmental signals, with a view to eventually elucidating their genetic control.

**Figure 2:**
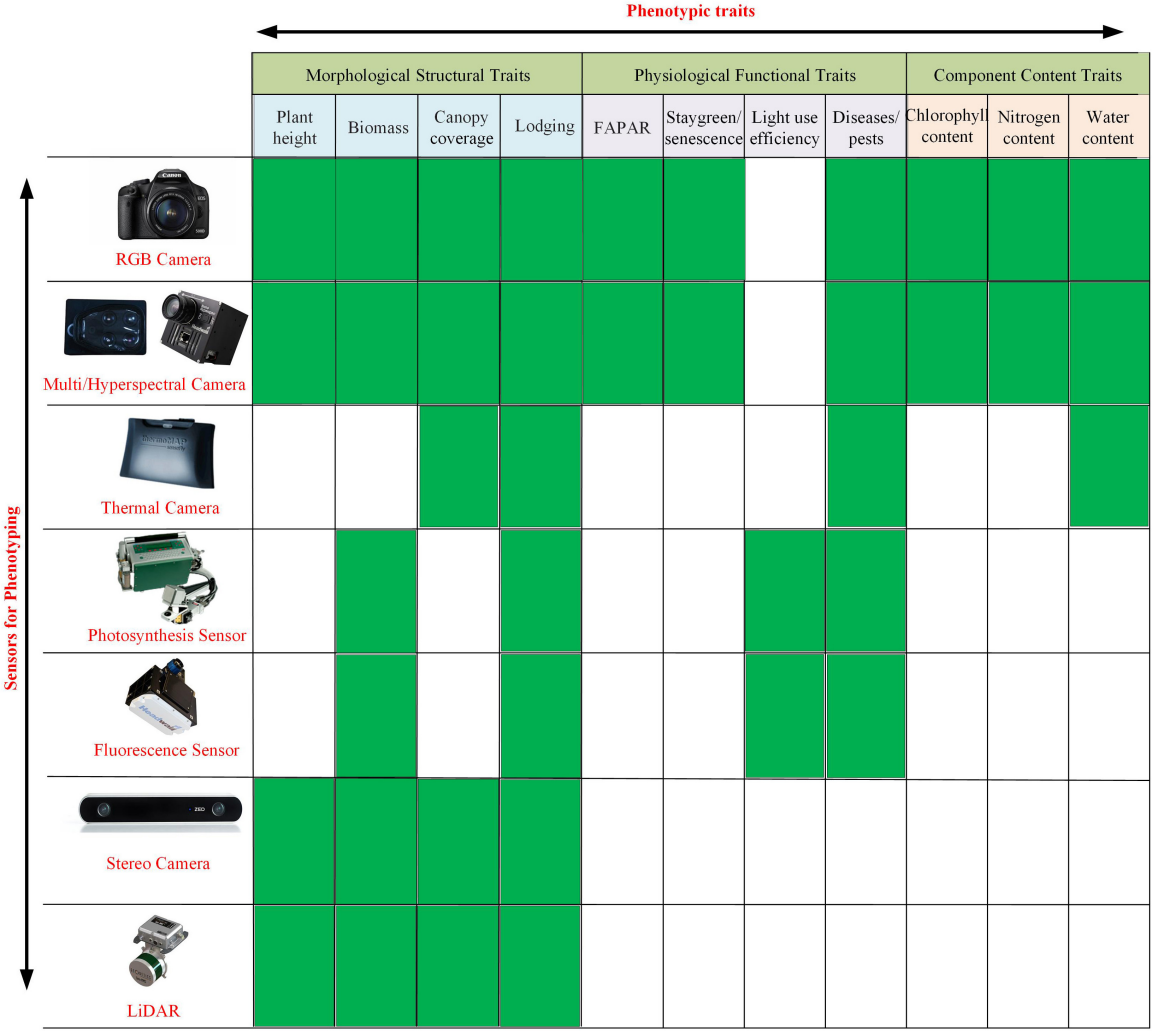
Sensors currently available to monitor, quantify, and estimate key morphological structural traits (e.g., plant height, biomass, canopy coverage, lodging), physiological functional traits (e.g., FAPAR, staygreen/senescence, light-use efficiency, disease/pests), and component content traits (e.g., chlorophyll content, nitrogen content, water content) of plants.

Given the mechanical structure of the platform and movement mode between the sensors and plants, an indoor HT3P can be categorized as either a benchtop type or a conveyor type. Table 1 shows specific examples and details of these two types of HT3P. No matter which type it is, the phenotyping platform integrates common cameras, supplemental light sources, automatic watering, and weighing devices, to automatically collect plant phenotypic data. Available cameras include those capable of capturing RGB, infrared (IR), fluorescence (FLUO), near-infrared (NIR), multispectral, or hyperspectral images. For example, the FLUO imager is used to obtain chlorophyll or photosynthesis-related characteristics (Choudhury et al., 2019). Hyperspectral imaging in particular provides access to crucial metrics, such as those for photosynthesis, chlorophyll, and nitrogen content. See Figure 2 for detailed information on the diverse sensors now available to monitor, quantify, and evaluate key agronomic traits.

**Table 1:** Overview of HT3Ps used in greenhouses and growth chambers under environmentally controlled conditions.

**Indoor HT3P**	**Model**	**Sensors**	**Throughput (pots)**	**Plants**	**Traits**	**Location**	**References**
Conveyor type	LemnaTec Scanalyzer 3D	RGB, NIR, FLUO	312	Barley	Biomass, plant height, width, compactness, drought stress	Germany	Chen et al., 2014; Neumann et al., 2015
	LemnaTec Scanalyzer 3D	RGB, NIR, FLUO, hyperspectral	672	Sorghum, maize, barley	Biomass, leaf water content	USA	Miao et al., 2020
	LemnaTec Scanalyzer 3D	RGB, NIR, FLUO, hyperspectral	2,400	Chickpea, wheat	Nutrient stress, salt stress, water content, nitrogen content	Australia	Neilson et al., 2015; Atieno et al., 2017; Bruning et al., 2019
	Bellwether	RGB, NIR, FLUO	1,140	Setaria	Plant height, biomass, water-use efficiency, water content	USA	Fahlgren et al., 2015a
	–	Hyperspectral	100	Maize	PLA, NDVI, perimeter, major axis length, minor axis length, eccentricity	USA	Ma et al., 2019
	HRPF	RGB, CT	5,472	Rice	Drought stress, tiller number	China	Yang et al., 2014; Duan et al., 2018
Benchtop type	Phenovator	Monochrome	1,440	*Arabidopsis thaliana*	PLA, PSII efficiency	The Netherlands	Flood et al., 2016
	Phenoscope	RGB	735	*Arabidopsis thaliana*	Rosette size, expansion rate, evaporation	France	Tisne et al., 2013
	–	RGB	350	*Arabidopsis thaliana*	Radiation dosage stress, projected area, convex hull area, perimeter length	Korea	Chang et al., 2020
	Phenoarch	RGB	–	Maize	Growth rate of ear and silk	France	Brichet et al., 2017
	Glyph	RGB	120	Soybean	Water use efficiency, drought stress	Argentina	Peirone et al., 2018
	LemnaTec Scanalyzer HTS	RGB, FPUO, NIR	–	*Arabidopsis thaliana*	Water stress	USA	Acosta-Gamboa et al., 2017

Compared with field conditions, although indoor experiments cannot provide the authenticity of soil system and the complexity of biological and abiotic stress for plants, the purpose of indoor HT3P experiment is to study qualitatively or quantitatively the response of representative or interesting plants to specific environment. Environmental control platform avoids the unpredictable phenotypic variation caused by the interaction between genotype and natural environment (G × E). Therefore, considering uncontrollable factors in the field, HT3P deployed in greenhouse or growth chamber is widely used to study the response of plants to specific growth conditions, and accurately capture the morphological structural, physiological functional or component content phenotypic indicators.

### Conveyor-Type Indoor HT3P

The conveyor-type HT3P operates in the “plant-to-sensor” mode. Potted plants are transported into an imaging room with cameras, passing through an automatic door on the conveyor that is controlled by computer for automatic imaging, after which plants are returned to their original growth positions. Cameras are typically installed on the top and side of the darkroom to perform this imaging, and/or the plants are rotated for data acquisition. The automatic door eliminates the interference of ambient light, and there are halogen lamps to provide illumination.

Scanalyzer 3D, a typical conveyor-type HT3P developed by LemnaTec GmbH (Aachen, Germany), has been adopted by some international organizations, covering the following versions (Yang et al., 2020). The Plant Accelerator of the Australian Plant Phenomics Facility (APPF) is a leading international plant phenotyping research institution. Its conveyor HT3P can handle 2,400 plants and is equipped with multiple imaging stations (RGB, NIR, FLUO, and hyperspectral), and this has been used successfully to study the nutrient deficiency of crops (Neilson et al., 2015) and salt tolerance of chickpea (Atieno et al., 2017). The four imaging chambers are separated and function independently of each other. Recently, Bruning et al. (2019) used just two hyperspectral imagers in its hyperspectral imaging room to evaluate the concentration and spatial distribution of water content and nitrogen level in wheat. In another example, the conveyor belt system in the Smarthouse (APPF, University of Adelaide) was used to study the effects of zinc (Zn) and an arbuscular mycorrhizal fungus upon tomato (Brien et al., 2020).

Similarly, Scanalyzer 3D, in the Greenhouse Innovation Center of University of Nebraska-Lincoln, allows the phenotyping of 672 plants with a height of up to 2.5 m, being able to collect RGB, FLUO, IR, NIR, and hyperspectral images from the top and side view of plants (Choudhury et al., 2016). Each imaging room is equipped with a rotating elevator that permits 360 side-views of a given plant (Choudhury et al., 2018). There are three watering stations with balance, which can apply watering to meet the target weight of the pot or in specific volume, for which the amounts of water added are recorded. Since the imaging chamber is self-contained, this HT3P unit allows the employed sensors to be adjusted according to research needs. For example, RGB, NIR, and FLUO cameras are used to analyze the spatiotemporal biomass accumulation of barley under drought stress (Neumann et al., 2015). In studying maize, Ge et al. (2016) used RGB and hyperspectral imaging rooms to analyze this crop's growth and water-use dynamics, in addition to quantifying its leaf water content. To measure the nutrient concentration and water content of plants, Pandey et al. (2017) relied solely on the hyperspectral imaging room of the Scanalyzer 3D, this being the first time hyperspectral data was used to detect the nutrient content of living plants *in vivo*. Furthermore, Miao et al. (2020) segmented the generated hyperspectral images of sorghum and maize, at the organ level, to identify genetic associations, which let them measure plant properties more broadly.

The bellwether phenotyping platform, at the Donald Danforth Plant Science Center, including a Convion (Winnipeg, Canada) growth chamber and an imaging station (LemnaTec Scanalyzer) (Fahlgren et al., 2015a). Plant barcodes on the pots are used for radiofrequency identification (RFID), to match up image data with the metadata. The 180-m-long conveyor belt system can accommodate 1,140 plants, which are transported into FLUO, VIS, and NIR imaging stations through dark adaptation channels. Interestingly, this conveyor system is divided into four modules that can run independently, or as a whole, which increases the research flexibility and scope of potential experiments. To sum up, as a widespread conveyor-belt HT3P for large-sized plants, Scanalyzer 3D is effective in studies of plant biology and plant breeding.

The purpose of the greenhouse is to provide a uniform, controlled environment. But since most conveyor HT3Ps often need to transport plants to the specific imaging room, this introduced microclimatic heterogeneity likely influences the plants' growth and response to environmental changes, rendering the phenotypic data collected inaccurate. Fortunately, the HT3P built by Purdue University overcomes this interference of a differential microclimate (Ma et al., 2019), in that plants are grown on cyclic conveyor belts throughout their whole growth cycle, thus exposing them to the same heat and radiation conditions. Huazhong University of Science and Technology and Huazhong Agricultural University (Wuhan, China) jointly developed a high-throughput rice phenotyping facility (HRPF) with an image analysis pipeline, able to perform color imaging and X-ray computed tomography (CT); it can monitor 15 agronomic traits of 1,920 rice plants (Yang et al., 2014). This HRPF was used to quantify the dynamic response of rice to drought (Duan et al., 2018). However, the investment cost of conveyor HT3Ps is high, and further improvement is needed to enhance flexibility.

The CT platform can high-throughput visualize and quantify external and internal geometric features, which offers the opportunity to collect morphological and anatomical characteristics of plants. There are two general types of CT platforms used in plant sciences: industrial CT scanners and medical CT. Industrial CT scanners with a higher resolution than medical CT, also known as micro-CT, μCT, or nano-CT, can be applied for the subtle phenotypic traits of plants. For example, Tracy et al. (2017) used μCT scanning to obtain detailed three-dimensional phenotypes of *Arabidopsis thaliana* and barley, which allows the measurement of spike size and further accurate staging at the flower and anther stages. This rapid and non-destructive method overcomes the traditional tedious steps in cytological microscopy, such as fixation, sectioning, staining, and microanalysis. Compared to μCT, medical CT can faster scan larger samples and larger numbers of samples, despite its lower resolution. Gomez et al. (2018) used a medical CT platform to study the geometric characteristics of sorghum stem, finding that medical CT estimates were highly predictive of morphological traits and moderately predictive of anatomical traits.

The conveyor-type HT3P (excluding CT) can carry samples of large size (e.g., sorghum and corn) and large capacity, but it may affect those plants with fragile stems due to shaking of the belt. And since spectral information is not collected *in situ*, there are environmental differences between the plants' growth location and the imaging room, which may lead to inaccurate phenotypic data. The CT platform enables the acquisition of meticulous morphological and anatomical traits of plant, which has great application prospects. The future conveyor-type HT3P will aim for high operational stability and environmental homogeneity, providing smooth plant transportation and accurate climate control for plant science research, and enabling complete precise phenotyping of plant traits throughout reproductive period.

### Benchtop-Type Indoor HT3P

In measuring phenotypic traits susceptible to environmental changes (temperature, wind, to name a few), especially for small species with fragile stems, it is a wise choice to keep plants still while the sensors are moving about. This is exactly how a benchtop HT3P works, for which the operation mode is one of “sensor-to-plant.” The imaging head is integrated with multiple sensors, driven by a computer-controlled mechanical arm, which automatically locates the position where a plant is growing and collects its phenotypic data *in situ*. In general, the benchtop HT3P also features a precisely controlled irrigation and weighing system, with supplemental light sources.

*Arabidopsis thaliana* is a prime model plant because of its wide distribution, fast life cycle, and relatively small genome (Kaul et al., 2000), making an ideal study species for the benchtop HT3P. The platform for measuring photosynthetic parameters (PSII, photosystem II), named Phenovator, can accommodate 1440 *A. thaliana* plants (Flood et al., 2016). Driven by an XY camera-movement system, the imaging head carrying the cameras measures photosynthesis and projected leaf area (PLA) at eight wavelengths, *via* an eight-position filter wheel installed on the monochrome camera. For the Phenoscope platform, its imaging system consists of digital camera only (Tisne et al., 2013), but it can carry 735 individual pots. Its ingenious feature is that it can continuously rotate each pot, so the sampled plant experiences the same external conditions, thus minimizing micro-environmental variation at the individual plant level and providing high spatial uniformity.

Similarly, the LemnaTec Scanalyzer HTS is equipped with a robotic arm that houses VIS, FLUO, and NIR cameras to take top views of small plants. It is has been used to study the time-dependent effect of water stress on *A. thaliana* (Acosta-Gamboa et al., 2017). Although the number of samples it can process is relatively small, it provides sufficiently rich spectral information. In mutation breeding, the phenotype discovery of many mutants is rapidly posing a limitation to molecular plant physiology research (Fraas and Lüthen, 2015). Recently, Chang et al. (2020) collected the growth images of 350 *A. thaliana* and compared the subtle morphological effects of different radiation dosages during its growing period, obtaining not only dynamic growth behavior information (such as the plant growth rate post-radiation) but also the phenotypic characteristics of dose effects.

Plant silk is normally difficult to detect and quantify because of its unique features. To overcome this, Phenoarch (INRA, Montpellier, France) made a breakthrough, when Brichet et al. (2017) used it to monitor the growth dynamics of corn ears and silks. That HT3P unit has two imaging cabins. First, the plants are rotated at a constant speed, and RGB cameras determine the spatial coordinates of the ears on them. Then the robotic arm assists in the automatic positioning of the camera at a 30-cm distance from the ear, to continue collecting of high-resolution images of silks. In this way, the daily growth of ear and silk of hundreds of plants can be tracked. Crop 3D, developed by the Chinese Academy of Sciences (Guo et al., 2016), takes LiDAR as the core sensor and integrates a high-resolution RGB camera with a thermal and hyperspectral imager, applying one key trigger to synchronously acquire multi-source data and extract plant morphology parameters. Specifically, the sensors adopt a vertical downward or overhead mode, to mount and shoot, carrying out single row scanning, multi-row scanning, and fixed-point positioning scanning.

Although a low-cost and non-commercial platform has a small sample capacity, it could also generate high-throughput phenotypic data. Glyph, as a representative, consists of four bridge-like structures, whose drip irrigation equipment and digital camera form a gantry that moves on the track between pair of rows. It has been successfully used for predicting the field drought tolerance in soybeans (Peirone et al., 2018). The SITIS platform, which consists of PVC pipes with irrigation points, was used to evaluate water stress tolerance of cotton cultivars (Guimarães et al., 2017), and it also enables the evaluation of plant roots. However, this platform is not an image-based, non-destructive one to measure plant traits, so it still requires much manual operation and experimental processing. The human-like robotic platform is a newer method to measure plant phenotypic traits instead of doing such manual operations. The *vivo* robotic system, consisting of a four degree of freedom (DOF) manipulator, a time-of-flight (TOF) camera, and a gripper integrated with an optical fiber cable and thermistor, can be used for the automatic measurement of maize and sorghum leaf traits (Atefi et al., 2019). More specifically, the TOF camera acts as the vision system, and the gripper can measure VIS-NIR spectral reflectance and temperature; however, its capture speed, as well as its capture success rate (78% for maize and 48% for sorghum), need further improvement. The rapid development of such a robot system can provide reference data and supplementary support for image-based plant phenotyping.

Strictly benchtop-type HT3Ps tend to focus on model plants of small size (e.g., *A. thaliana*) and allow for the collection of trait data associated with subtle phenotypic changes, and their situ extraction also ensures homogeneity of growth environment and undisturbed development. However, sophisticated commercial HT3Ps tend to be capital-intensive, while low-cost self-developed platforms have small sample capacity and low throughput, whose quality, credibility, and abundance of phenotypic data can be somewhat reduced. Fortunately, artificially intelligent plant growth chambers, plant factories, and rapid iterative breeding have opened new avenues for indoor HT3Ps. The future benchtop-type HT3P may be able to omnidirectionally monitor, capture, and track subtle morphological and physiological changes of multi-level traits in a wide range of model plants, with high-throughput and full-automation, to reveal functional gene expression and biogenetic regulation patterns.

## HT3P For Field Phenotyping In Notoriously Heterogeneous Conditions and Relatively Uncontrollable Environmental Factors

Plants that grow naturally in the field are affected by weather (e.g., rain, frost, snow), biotic and abiotic stresses (e.g., drought, low-temperature, low-nitrogen, pests), as well as soil properties (e.g., nutrient gradients, heterogeneity, micro-environment), all of which are extremely distinct from the environment of greenhouse and growth room, making the field crop phenotype an intricate one. Whereas controlled environment, image-based phenotyping platforms are almost universally popularized worldwide, the majority of crop breeding appears in the field with little if any selection in controlled environments (Furbank et al., 2019). And the indoor environment can only simulate but not recreate the real field setting. These factors stimulated the exponential increase of a wide variety of HT3Ps in fields.

**Figure 3:**
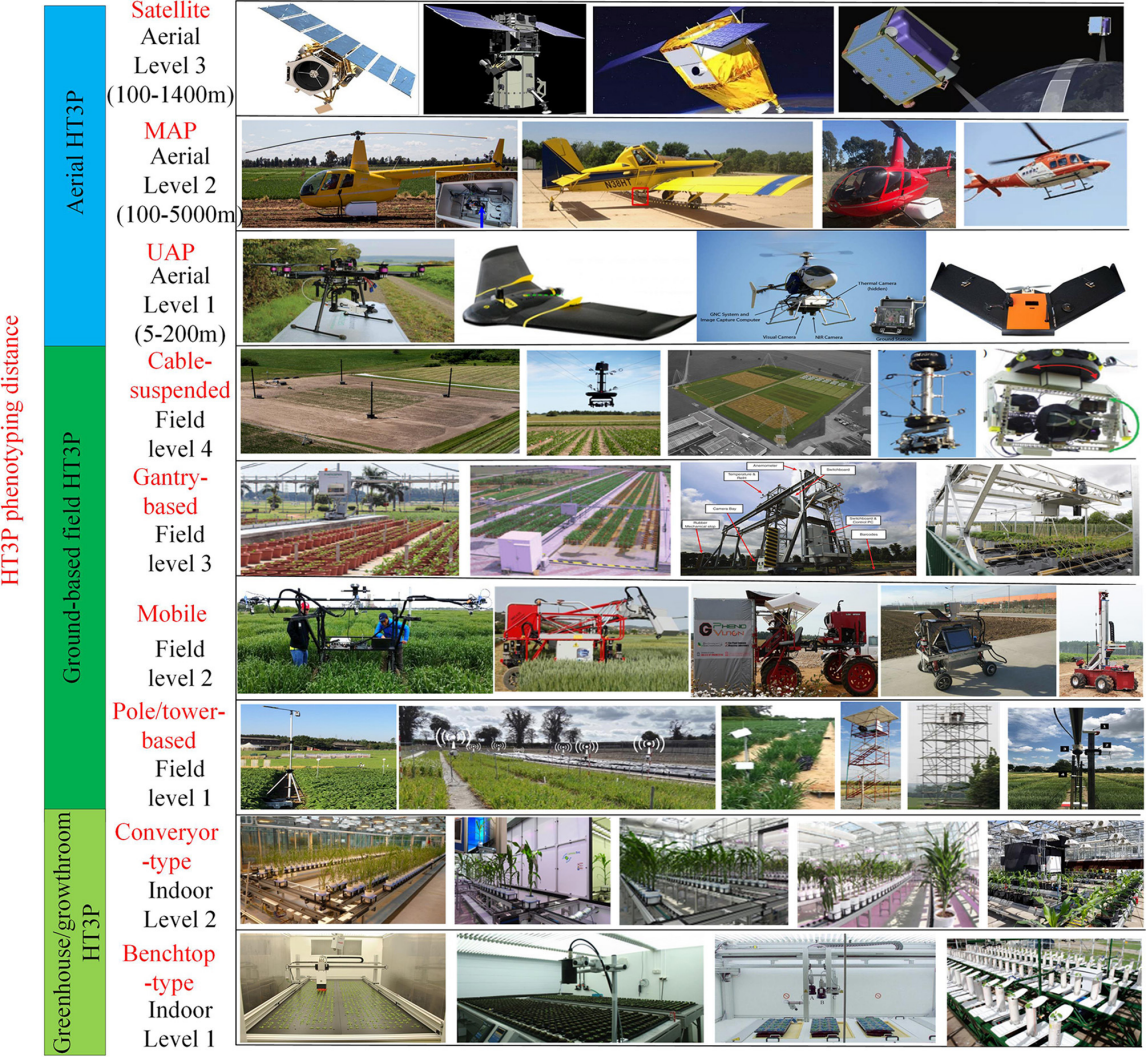
The various types of HT3Ps mentioned in this review (partial display) including HT3Ps in the greenhouse/growth chamber (i.e., benchtop-type and conveyor-type), field ground-based HT3Ps (i.e., pole/tower-based, mobile, gantry-based, and cable-suspended) and aerial HT3Ps (i.e., UAP, MAP, and satellite).

Apparently, field HT3Ps operate in the “sensor-to-plant” mode. According to their usage scenarios and imaging distance, field HT3Ps can be categorized into ground-based and aerial platforms. The relationship between a platform's characteristics and field crop traits determines the efficiency of the phenotype platform to a certain extent (Kuijken et al., 2015). Based on this, ground-based platforms can be further classified as pole/tower-based, mobile, gantry-based, and cable-suspended. Likewise, aerial platforms could be categorized further, as the unmanned aerial platform (UAP), manned aerial platform (MAP), and satellite platform. Figure 3 shows specific scenarios of various types of applied HT3Ps.

Despite the complex interactions of genotype, environment, and management (G × E × M), the proliferation of a wide variety of HT3Ps in recent years has greatly assisted researchers in understanding the genetic structure of crops, obtaining high-quality genetic gains, and improving the ability to genetically analyze crop traits related to yield and stress resistance. However, the diversity of HT3Ps also brings with considerations of availability, feasibility, standardization, big data, and reliability.

### Ground-Based Field HT3P

Ground-based HT3P means proximal phenotyping that can provide higher resolution data than aerial remote sensing. It is convenient to collect phenotypic data of time series and analyze the dynamic response and time dependence of phenotypes. However, the ground-based HT3P is not suitable for large-scale phenotyping tasks. Table 2 shows specific examples and details of these four types.

**Table 2:** Overview of ground-based HT3Ps used in the field under real uncontrolled environmental conditions.

**Field HT3P**	**Designation**	**Sensors**	**Feature**	**Plants**	**Traits**	**Country**	**References**
Pole/tower-based	–	Terrestrial laser scanner	Maximum load of 50 kg, 3.8 m high, covers 120 m	Maize, soybean, wheat	Canopy height	Switzerland	Friedli et al., 2016
	CropQuant	RGB, NIR	Combined with IoT	Crop	Crop growth rate	UK	Zhou et al., 2017
	PhenoCam	RGB	Large phenotyping network	Ecosystem	Canopy greenness	USA	Richardson et al., 2018
	–	Hemispherical video camera	Automatic camera track system	Wheat, oat, barley	Crop lodging	USA	Susko et al., 2018
	–	Laser-Induced Fluorescence Transient (LIFT)	Covers 50 m	Barley, Sugar Beet	Photosynthesis	Germany	Raesch et al., 2014
	–	RGB, NIR	Consists of two 8-m high towers	Rice	Shoot biomass, panicle number, grain weight	Colombia	Naito et al., 2017
Mobile	–	LiDAR, RGB, thermal IR, IR thermometer, hyperspectral	Speed of 1 m/s	Wheat	Canopy height, leaf angular distribution, leaf area, leaf volume, spike number, VIs, canopy transpiration	Australia	Deery et al., 2014
	–	Ultrasonic, NDVI, thermal IR, spectrometers, RGB	A “stop-measure-go” model	Soybean, wheat	Canopy height, NDVI, canopy temperature	USA	Bai et al., 2016
	Phenomobile Lite	LiDAR, RGB, NDVI	Three-wheeled buggy	Wheat	Plant height, biomass, ground cover	Australia	Jimenez-Berni et al., 2018
	GPhenoVision	RGB-D, thermal, hyperspectral	Modularity, customizability	Cotton	Canopy height, width, growth rate, projected leaf area, volume, yield	USA	Jiang et al., 2018
	–	LiDAR	Group observation on parcel, “stop-measure-go” model	Maize	Plant height	China	Qiu et al., 2019
	–	Ultrasonic, spectrometer, RGB, IR radiometer	Emergency stop and inspection	Soybean	Canopy height, canopy coverage, NDVI	USA	Murman, 2019
Gantry-based	LeasyScan	Planteye	Continuous phenotyping	Peanut, cowpea, pearl millet, maize	Canopy transpiration, plant height, 3D leaf area, water use efficiency	India	Vadez et al., 2015; Sunil et al., 2018
	Phénofield^r^	RGB, VIS-NIR, LiDAR	Conditions controlled in the field	Wheat	Water stress resistance, nitrogen stress resistance	France	Beauchene et al., 2019
	Field Scanalyzer	RGB, FLUO, thermal IR, hyperspectral, 3D laser scanner	Fully automatic or remote manual operation	Wheat	Canopy height, spike number, canopy closure, canopy temperature, NDVI, photosynthesis	UK	Virlet et al., 2017
	Mini-Plot	Hyperspectral	Divided into open field and closed greenhouse areas	Barley	Disease severity	Germany	Thomas et al., 2018
Cable-suspended	NU-Spidercam	Multispectral, thermal IR, LiDAR, VIS-NIR spectrometer	Covered 0.4 ha, 30-kg payload, maximum speed of 2 m/s	Soybean, maize	Plant height, ground cover, canopy temperature	USA	Bai et al., 2019
	FIP	Spectrometer, ultrasonic, DSLR, thermal, laser scanner, operator camera	Covers 1 ha, 2 to 5 m above the canopy, 12-kg payload, maximum speed of 2 m/s	Winter wheat, maize, soybean	Canopy cover, canopy height	Switzerland	Kirchgessner et al., 2017

#### Pole/Tower-Based Field HT3P

The pole/tower-based HT3P is formed when sensors are mounted directly atop a pole or tower made of aluminum, steel, or plastic fibers, which can be of stationary or mobile type. Although this platform is simple in structure, similar to a small weather station, high throughput and complexity are not necessarily synonymous.

Friedli et al. (2016) applied a pole-based, terrestrial laser-scanning (TLS) platform, to monitor canopy height growth in maize, soybean, and wheat. The TLS is done *via* a laser scanner mounted upside down on a 3.8-m high aluminum elevator tripod. Its time and spatial resolution depend on the crop variety assessed and scanning distance. Combined with “Internet of Things” (IoT), CropQuant is equipped with RGB and No Infrared cameras to continuously monitor crop growth through high-resolution time-lapse photography (Zhou et al., 2017). It can be powered by batteries and solar panels and connected to an in-field WIFI network, as a mesh network node, to form an IoT-mode HT3P that quantifies crop growth and development. Furthermore, PhenoCam is a large phenotyping network, consists of a series of widely deployed digital cameras that automatically capture RGB images (typically, at 30 min intervals) to track biomes' vegetation phenology (Richardson et al., 2018). The camera is mounted on a pole, mast, or building. Although the data source is only visible images, such massive time series data sets can monitor the dynamic changes of an ecosystem.

A mobile handheld pole-based, Phenocorn, is integrated with a GreenSeeker (portable device), an IR thermometer, a web camera, and a global positioning system (GPS) receiver (Wei, 2017). This device can simultaneously collect normalized vegetation index (NDVI) and canopy temperature. Although the 12 kg hand-held Phenocorn can be carried on a person's back, it still requires much manual labor. Fortunately, it can modified for use in a cart that is manually pushed to collect phenotypic traits in the field (Crain et al., 2016). As is well-known, it is quite difficult to capture, track, and quantify crop lodging and crop movement. In tackling this, Susko et al. (2018) developed an automatic camera-tracking HT3P that consisted of a hemispherical video camera, a computer, and an industrial curve track system; its motor-driven camera moves along the track for phenotypic data acquisition. What makes this breakthrough so novel is that it can be used for dynamic imaging in video or static frequent imaging, thus allowing for the study of new plant phenotypes.

The tower-based HT3P is similar to the stationary pole-based platform, with sensors installed atop the tower, but the dimensions and height of a tower-based platform are generally higher and larger than that of pole-based. In a study on photosynthetic efficiency of barley and sugar beet, laser-induced fluorescence transient (LIFT) instruments were placed on the top of a 10-m high scaffold to measure the photosynthetic performance of agroecosystems (Raesch et al., 2014). However, the LIFT signal in the target area introduced noise from plant stems and the soil. In later work, Naito et al. (2017) installed an improved multispectral single-lens imaging system (i.e., VIS and NIR cameras), on two 8-m high towers, which could collect crop images from eight angles to estimate rice yield-related traits. Their results showed that the system has great potential for yield estimation during early crop development.

Both pole-based and tower-based HT3Ps are easy and low-cost to build and maintain, and are convenient for temporary use and multi-site deployments to form networks. However, phenotypic area of coverage and spectral information are extremely limited for a single unit, and multi-site trials increase the cost for large-scale field experiments. Looking ahead, being portable, scalable, rotatable, robust, and easy to install and remove are anticipated key features of pole/tower-based platforms, and phenotypic networks of economically-efficient distributed pole/tower-based HT3Ps will play a prominent role in the future of multi-site large-scale experiments and the calibration of high-dimensional phenotypic data.

#### Mobile HT3P

The mobile HT3P can move through the field and collect crop phenotypic traits in a semi-automatic or fully automatic manner, including refitted agricultural machinery (e.g., tractor, sprayer, or harvester), self-developed mechanical platform (e.g., cart or buggy), and commercial automatic platform. A mobile HT3P is generally composed of four subsystems: sensing system, data acquisition system, mechanical platform, and drive system. The sensing system covers GPS, environmental sensors (e.g., sunlight, wind speed), and phenotypic sensors (e.g., RGB, multispectral, hyperspectral, thermal). The data acquisition system generally is the data acquisition software on an onboard computer. Mechanical platform is for load-bearing and mobility, and the drive system can be classified as electricity, engine, and manpower. Theoretically, any agricultural mobile platform has the potential to be converted into a mobile HT3P.

The mobile HT3P converted from a tractor, sprayer, or harvester makes rational use of precision agricultural machinery that already exists. In the early stages, a pioneering field-based mobile was developed in Canberra's High-Resolution Plant Physics Facility (Deery et al., 2014). It was equipped with a height-adjustable sensor array, including LiDAR, RGB, thermal IR, and hyperspectral cameras. Driven by the mobile, the sensors pick up traits' data along the surveyed plots. A cherry picker installed a linear scanning bar with spectral cameras has also been applied as a mobile HT3P (Pinto et al., 2016); its imaging cameras aim to collect canopy radiation in a linear push broom mode. The temporal change of canopy photochemical activity was tracked by generating sun-induced chlorophyll fluorescence (SIF) map. A multi-sensor system developed by Bai et al. (2016) consists of five sensor modules, and this platform was used to measure canopy traits of soybean and wheat. They adopted the “stop-measure-go” mode, to ensure the sensors precisely aligned with the plots, collecting plot-level phenotypic data that were free of blurring. But to control such a HT3P, a certain number of operators must participate. GPhenoVision is assembled from a high-clearance tractor, carrying RGB-D, thermal, and hyperspectral cameras for quantitative assessment of cotton canopy growth and development (Jiang et al., 2018). Customizability and modularity are the two key characteristics of GPhenoVision, which allows researchers to rapidly develop and upgrade sensing modules with specific phenotyping purposes, reduces development effort when adding or removing modules, and prevents malfunction of the entire system. Jimenez-Berni et al. (2018) installed a LiDAR, an NDVI sensor, and a digital camera on a low-cost three-wheeled buggy, called Phenomobile Lite, to evaluate canopy height, ground cover, and aboveground biomass. However, to change its direction, a “stop” button is need to be manually pressed, and almost the whole process requires operator follow-up.

To achieve the automatic measurement of yield-related traits, Bao et al. (2014) presented a mobile HT3P that could automatically obtain stereo images of sorghum plants. It was retrofitted from a garden tractor, equipped with six stereo camera heads on a vertical pole, which can be triggered synchronously. Three-dimensional images of two rows of crops can be collected in a single pass. The drawback is that before each bout of data collection, the platform must be driven manually through the field, stopping, and recording each sampling point to generate paths. Later, an agricultural mobile robot mounted with a 360°-view LiDAR, developed by Qiu et al. (2019), was used to efficiently calculate the row spacing and plant height of a maize field. Compared with in-row and one-by-one phenotyping methods, the 3D laser scanner sitting atop this robot obtains group observation of parcels. While it moves in a “stop-and-go” manner, the phenotypic data of parcel-level plant group could be simultaneously collected. Unlike the wheeled mobile platform, Stager et al. (2019) employed a modified crawler robot to collect sub-canopy traits at low elevation. But branches, tillers, or roots may hinder the movement of such crawler robots. Automated robots offer the prospect of unattended field operations, which likely be a major focus of future research of agricultural phenotyping platforms.

By letting the phenotyping height of the mobile HT3P vary, to adapt to different growing stages of crops, Flex-Ro was developed to identify differences in the emergence and maturity stages among soybean varieties (Werner, 2016). PhenoBox of Flex-Ro mainly integrates data acquisition hardware, while its height-adjustable PhenoBar, located at the front of this mobile platform, mainly consists of three sensor units, capable of covering 4.5-m swath (Murman, 2019). An operator controls the machine through the remote box or a MATLAB application called FlexRoRun. It is worth mentioning that a 3D smart sensor is incorporated, for obstacle detection, which successfully detects pedestrian-sized objects and triggers parking. This will be a critical security consideration for future robotic HT3Ps. Likewise, a multi-purpose field robot in combination with various apps can achieve different functions. For example, Bonirob robotic platform with the phenotyping app, penetrometer app, and precision spraying app can monitor plant growth, measure soil parameters, and apply chemical weeding, respectively (Bangert et al., 2013). Unlike the mobile HT3P for small-sized or early-growing crops, Robotanist can navigate autonomously in the fields of tall crops, such as corn or sorghum (Mueller-Sim et al., 2017). Interestingly, it has a three DOF manipulator that can touch and measure the strength of plant stalks, which is a phenotypic innovation based on contact.

A semi-automatic self-made mobile HT3P can reduce the development cost and soil compaction (because of lightweight architecture), but normally requires one or more operators to follow-up (Bai et al., 2016, 2018; Jimenez-Berni et al., 2018). Moreover, because of the “stop-measure-go” mode and slow response speed of low-cost sensors, the efficiency of crop traits' data acquisition cannot be guaranteed. Concerning the mobile HT3P based on the modified tractor, sprayer, or harvester, it often needs special personnel to drive. Its large volume and weight risk causing soil compaction and mechanical disturbance to the crops, which precludes the deployment in the field. And the faster travel speed than self-made cart also may not guarantee the quality of phenotypic data obtained. But the payload is large, so it can integrate diverse sensors to collect multi-source information. Robotic HT3P is capable of automatically navigating through the field and collecting data on crop traits, as well as doing continuous phenotyping throughout the day and night, but its development and maintenance costs are expensive. The future mobile HT3Ps will likely aim for lightness, automation, modularization, and customization, and the mechanical arm and emergency braking mechanism will offer potential applications that could greatly improve the flexibility, autonomy, and security.

#### Gantry-Based Field HT3P

As a gantry frame is equipped with a sensor box, and moves along the track and collects crop traits along XYZ directions, it becomes the gantry-based HT3P. When it moves back and forth on the track, repeated phenotyping done this way avoids the possibility of soil compaction and damage of crops' normal development.

LeasyScan, equipped with a set of scanners (PlantEye F300, Phenospex, Heerlen, the Netherlands), can perform continuous, synchronous and automated monitoring of plant water use and leaf canopy development, *via* linear movement above the surveyed plants (Vadez et al., 2015). The trigger and stop of each measurement are controlled by a mechanical barcode, which is also used for distance calibration from the scanner to the ground. LeasyScan was also utilized in a pre-breeding, genetic resource identification experiment with hybrid maize (Sunil et al., 2018), in which it measured plant height, 3D leaf area (i.e., total leaf area), and leaf area index (LAI). Similarly, Virlet et al. (2017) applied Field Scanalyzer (LemnaTec) to monitor and quantify morphological traits of wheat organs and canopy. Through the control software in the master computer, the gantry-based HT3P can operate in the full-automatic or manual mode. Unfortunately, though it can sample continuously, 24 h per day, but no two sensors can collect images simultaneously. Then, Field Scanalyzer also was used to determine wheat canopy height and further validate the potential of functional mapping analysis for detecting persistent quantitative trait loci (QTLs) (Lyra et al., 2020).

A phenotypic experiment that precise management is involved in the sufficiently realistic field environment is ideal for deciphering G × E × M interactions. PhénoField^R^ is the first field-based facility in the European Union, one equipped with high-throughput phenotyping devices on a gantry, automatic irrigating mobile rainout shelters on tracks, and environmental recording sensors. By controlling both irrigation and fertilization, this gantry-based HT3P was used to investigate water and nitrogen stress (Beauchene et al., 2019). Likewise, Mini-Plot, developed by Forschungszentrum Jülich, consists of a closed greenhouse area and an open-fenced area, containing 90 and 30 Mini-Plots, respectively; it was used to quantify the disease severity of barley varieties (Thomas et al., 2018). The measuring head consisting of a hyperspectral sensor, a mirror-based scanning system, and an automatic positioning system moves over the plant for imaging. Such gantry-based HT3Ps for phenotyping, which can integrate genetics, field environmental factors, and management practices, has greatly enhanced the understanding of genetic control pattern for breeders and plant biologist.

However, once the construction and installation of the gantry-based HT3P is completed, multi-site experiments can be expensive or even inoperative. High development, operation, and maintenance costs are also several factors that researchers should be aware of and bear in mind. Fortunately, gantry-based HT3Ps with a high degree of automation can collect time-series phenotypic data of high resolution. Owing to its large payload and continuous operation ability, which greatly improves expansibility, this approach does provide an opportunity to better understand the dynamics of plant circadian rhythms. Extending phenotypic area, shrinking volume, and reducing cost will imbue the gantry-based HT3Ps with better application prospects.

#### Cable-Suspended Field HT3P

A cable-suspended HT3P is mainly composed of sensing system, data acquisition system, mechanical transmission system, and drive system. Sensing system and data acquisition system are generally integrated in a sensor bar. Winches, cables, poles, and pulleys make up the drive and mechanical transmission system. Four poles are distributed in four corners of the field and a winch house for controlling the cables sits at the bottom of the pole. The sensor array with a GPS unit can be precisely positioned above the interested region for traits' data acquisition through cable-driven.

A typical representative of cable-suspended HT3P is the “FIP,” located at the ETH research station in Switzerland. Its sensor heads integrated with a DSLR camera, a laser scanner, and a thermal camera can collect crop phenotypic data from 2 to 5 m above the canopy. The feasibility of FIP in monitoring canopy coverage and canopy height of winter wheat, maize, and soybean was verified by Kirchgessner et al. (2017). Furthermore, the Nu-Spidercam at the University of Nebraska has a multi-sensor systems, a subsurface drip irrigation (SDI) system and an automatic weather station (Bai et al., 2019), which is capable of accurately and flexibly capturing crop traits, such as plant height, canopy cover, and spectral reflection. Its sensor bar integrates numerous sensors, so software is designed for selecting available sensors to meet particular experimental needs. A drawback is that it only can operate continuously for 6–8 h when fully charged.

Phenotyping sensors of the cable-suspended HT3P is performing tasks over the crop canopy by cables during data collection of agronomic traits, meaning that the cable-suspended HT3P has lower dependence on soil conditions and less interference to plants than mobile HT3P. Phenotypic traits information of time series can still be collected within the established crop growth-monitoring period. And it can cover larger phenotypic region but has lower load than do gantry-based HT3P. In general, field HT3Ps are considerably weather-dependent and region-limited. Allowing stabilized imaging of plants clusters and continuous monitoring of crop growth at low elevation for a longer duration will be a likely future trend for cable-suspended HT3Ps.

### Aerial HT3P Vulnerable to Weather Constraints and Aviation Regulatory

According to the imaging distance employed, aerial HT3Ps encompass the Unmanned Aerial Platform (UAP), Manned Aerial Platform (MAP), and satellite platform. UAP usually requires an operator at a ground station to remotely control or execute the flight task, by following a set planned path. Crop images are automatically acquired by onboard sensors. Nevertheless, MAP needs a dedicated person to pilot the aircraft and another passenger to manually capture the crop images while in flight. UAP and MAP typically carry global navigation satellite system (GNSS), but often this must be coupled with ground control points (GCPs) and calibration boards, for accurate georeferencing of the acquired data and for correcting its spatial resolution. A satellite platform collects large-scale field images from space, letting one access and download this satellite data as needed. Table 3 lists the specific applications and details of the aerial HT3Ps. When compared with a ground-based platform, an aerial HT3P can collect broader-scale phenotypic regions in a shorter period. However, the aerial platform has strong weather dependence and relatively low spatiotemporal resolution because of its flight altitude and imaging distance.

**Table 3:** Overview of aerial HT3Ps subject to air regulatory regime and weather constraints.

**Aerial HT3P**	**Designation**	**Sensors**	**Flight altitude (m)**	**Plants**	**Traits**	**References**
UAP	Phantom 4	RGB	20	Inbred lettuce lines	Carotenoid content	Mascarenhas Maciel et al., 2019
	3DR Solo quadcopter	Multispectral	45, 50	Maize	NDVI, chlorophyll red-edge index (CHL), hemispherical-conical reflectance factors (HCRF)	Fawcett et al., 2020
	Customized	Hyperspectral	80	Winter barley	NDVI, yield	Oehlschläger et al., 2018
	Self-developed octorotor	RGB, multispectral	25	Rice	Canopy height, VIs, canopy coverage	Wan et al., 2020
	Matrice 600 Pro	RGB, multispectral camera, infrared thermal	50	Cotton	Yield	Feng et al., 2020
	Tuffwing Mapper	RGB	120	Sorghum	Plant height	Han et al., 2018
	Ebee	Multispectral	50	Wheat	Yield	Hu et al., 2020
	Self-developed	Multispectral, thermal	150	Maize	Low-nitrogen stress resistance	Zaman-Allah et al., 2015
	Anaconda	RGB, multispectral	120	Sorghum, maize	Plant height, VIs	Shi et al., 2016a
MAP	Robinson R44 Raven helicopter	Radiometrically-calibrated thermal	60, 90	Wheat	Canopy temperature	Deery et al., 2016
	Air Tractor AT-402B	RGB	152–3,048	Crop	Pest severity	Yang and Hoffmann, 2015
	–	LiDAR	1,500	Maize	Biomass	Li et al., 2015
Satellite	GeoEye-1	Multispectral	684 k	Turfgrasses	Nitrogen content	Caturegli et al., 2015
	RapidEye	Multispectral	630 k	Wheat	Nitrogen stress	Basso et al., 2016
	Sentinel-1 and RADARSAT-2	Synthetic aperture radar (SAR)	700, 798 k	Wheat	Crop height, angle of inclination	Chauhan et al., 2020
	Fluorescence explorer (FLEX)	Fluorescence Imaging Spectrometer (FLORIS)	814.5 k	Terrestrial vegetation	Photosynthesis	Drusch et al., 2017

#### Unmanned Aerial Platform (UAP)

In recent years, due to the reduction of Unmanned Aerial Vehicle (UAV) prices and the relaxation of air traffic regulations, the application of UAP in agricultural research has increased exponentially. This so-called UAP is a kind of aerial platform, for which UAV is the carrier, that integrated onboard sensors, a GPS unit, an inertial measurement unit (IMU), a battery and a crucial gimbal—for correcting the influence of pitch and roll motion—to collect phenotypic data at the plant canopy scale. To collect high-precision geographic position of plots, both GCPs and calibration boards are needed. Its successful phenotyping of plants depends on the characteristics of UAV and the properties of the deployed sensors (Sankaran et al., 2015). According to its most distinguishing feature, UAPs can be classified as multi-rotor or fixed-wing. UAV's flight, however, is weather-dependent, and the ideal conditions are clear, windless, and dry weather, similar to those required when applying agronomic inputs.

Because of its limited payload and endurance, UAP can only carry a finite number of phenotypic sensors (generally, no more than 3). A multi-rotor UAV can be a quadcopter, hexacopter, or octocopter. Compared with its fixed-wing counterpart, it has lower flight altitude and slower flight speed, but is capable of vertical takeoff and landing (i.e., it can hover). With respect to the multi-rotor UAP, the use of one sensor is most common. For example, RGB images acquired by multi-rotor UAPs have been widely used for researching the growth rate of wheat (Holman et al., 2016), carotenoid levels of inbred lettuce lines (Mascarenhas Maciel et al., 2019), vegetation index (Buchaillot et al., 2019), wheat height (Villareal et al., 2020), growth of different maize inbred lines (Wang et al., 2019), and canopy extraction of orchard (Wu et al., 2020). Parrot Sequoia (Parrot, France) and Micasense RedEdge (Micasense, US) are the more familiar multispectral cameras used with UAP. For example, Parrot Sequoia was used to evaluate the accuracy and spatial consistency of hemispherical-conical reflectance factors (HCRF) (Fawcett et al., 2020). Notably, cameras are fixed (at a 3-degree angle) to offset the average forward tilt in flight. The recent declining cost of thermal cameras has made airborne thermography more widely used. In addition, ICI camera was proven to be better than either the FLIR or thermomap camera for evaluating plants' physiological and biochemical characteristics (Sagan et al., 2019a). However, atmospheric and emissivity calibration are remained challenges to thermal imaging. Likewise, hyperspectral sensors on the multi-rotor UAP were used to predict the yield of winter barley (Oehlschläger et al., 2018). Interestingly, for that, they used a mirror to guide the ground images into horizontally-positioned sensors. Compared with mere RGB data, collecting additional spectral data enables more robust predictions. In such a setting, RGB and multispectral sensors are integrated into the UAP, as was done to evaluate the yield of rice (Wan et al., 2020), plant height of sorghum (Kakeru et al., 2017), ground cover of cotton (Duan et al., 2017), and senescence rate of wheat (Muhammad et al., 2018). To obtain multi-source phenotypic data, three sensors are also employed on the multi-rotor UAP. For instance, an RGB camera, a multispectral camera, and an thermal IR imager were used together for cotton yield estimation (Feng et al., 2020). However, due to the limited payload, two independent flights of a multi-rotor UAP are occasionally required. For example, in assessing genotypic differences in durum wheat production, RGB images were obtained on the first flight and multispectral canopy information later collected on the second pass (Gracia-Romero et al., 2019).

Compared to the multi-rotor, a fixed-wing UAP has longer flight time, higher flight altitude, greater payload, and faster flight speed. But the fixed-wing UAP lacks hovering capability and has certain requirements for its takeoff and landing (e.g., a runway). The flight speed may cause blurred images, a problem resolved by using imaging sensors with high frame rate and short exposure time (Shi et al., 2016b). Although the fixed-wing UAP does have a relatively large payload, it is almost always relied on a single sensor. For instance, an RGB camera mounted on Tuffing Mapper (Tuffing LLC, Boerne, USA) was used to evaluate the plant height of sorghum (Han et al., 2018). The fixed-wing UAP has a semi-autonomous horizontal takeoff and landing (HTOL), controlled by the Pixhawk controller. Similarly, an RGB camera, with an internal infrared filter removed for color infrared (CIR) detection, was used to assess the height and crown diameter of olive trees (Díaz-Varela et al., 2015). The eBee UAV (senseFly, http://www.geosense.gr/en/ebee/) is becoming a commonly used fixed-wing platform. A multispectral camera mounted on eBee was used to evaluate the yield of early wheat genotypes (Hu et al., 2020) and thermal camera performance (Sagan et al., 2019a), as well as for identifying, positioning, and mapping weedy patches of *Silybum marianum* (Tamouridou et al., 2017). Research on this platform shows that using its highest resolution fails to provide the highest accuracy for weed classification. To assess the spatial variation of maize under low nitrogen stress, Zaman-Allah et al. (2015) developed a fixed-wing UAP with a multispectral camera and a thermal camera, controlled by an automatic navigation system. Fixed-wing UAPs have enough of a payload to carry three sensors. The Anaconda (ReadyMadeRC, Lewis Center, Ohio), fixed-wing UAP equipped with two multispectral cameras and a high-resolution digital camera, was used to collect phenotype data of corn and sorghum (Shi et al., 2016a), for which it employed the traditional configuration of a twin boom thruster. Interestingly, an external GPS unit was added to the digital camera for accurate positional information. Although the 3DR Pixhawk autopilot system has been applied for autonomous takeoff and landing, during its flight the Anaconda must be controlled manually. Ingeniously, the unmanned helicopter is an alternative type of UAP. For example, the Pheno-Copter (gas-powered) with a visible camera, a NIR camera, and a thermal IR camera was applied to measure sorghum ground cover, sugarcane canopy temperature, and crop lodging (Chapman et al., 2014). However, Pheno-Copter's flight needs to be controlled from a ground station, and this often requires specialized training.

If accurate geo-registration accompanies the image acquisition process of UAP, precise micro-plot extractions should be greatly improved (Hearst, 2014). To achieve this goal, he sensor configuration pattern must also undergo improvement. The data collected by UAP requires a GNSS and an inertial navigation system (INS) for spatial matching and georeferencing. To facilitate the calculation of the camera's actual geographic location corresponding to a given image acquisition, a more than 60% overlap of adjacent images is generally needed (Jin et al., 2017). In this context, the spatial offset (lever arm) and angle relationship (boresight angles) between the GNSS/INS unit and sensors are of great significance. Furthermore, compared with the lever arm, the boresight angle matters more. In view of this, Habib et al. (2018) recently employed a UAP, with push-broom hyperspectral sensors, to establish three boresight angle-calibration methods (around GCPs, tie points, and approximate means). While spatial positioning information may be obtained from an airborne GNSS/INS unit, its resolution is not high enough. Therefore, in order to carry out high-precision geographical referencing, establishing a proper layout of ground control points (GCPs) and calibration boards is necessary (Yu et al., 2016). Further, multifunctional GCPs were suggested for calibrating geometric and reflectance, and their usage significantly improved the phenotyping accuracy and also reduced manpower (Thomasson et al., 2019). Likewise, Han and Thomasson (2019) developed an automatic mobile GCP equipped with two RTK-GPS units, a navigation computer, and an integrated driving controller. It can reliably recognize and predict the behavior and activities of the UAP during the flight instead of traditional fixed GCPs, which explores the potential of improving the accuracy and efficiency of data collection.

Since the relevant airspace regulations remain strict, the huge potential of UAP cannot yet be fully exploited. Despite being susceptible to weather, payload, endurance, and aviation regulatory constraints, UAPs characterized by large-scale phenotyping, efficiency, flexible flight plans, and relatively low cost have gradually shifted the trend in phenotypic missions from the ground into the air. In recent years, with policy adjustment, hardware optimization, commercial UAVs' price reduction, advances in battery technology, and operation simplicity, UAP has come to fully display its outstanding abilities in plant phenotyping and plant science. Developing strategies for crop phenotype by remote sensing (Yang et al., 2017), improving performance such as endurance, payload, and stability, reducing the cost of sensors, and promoting data processing capabilities are the future trends of UAP.

#### Manned Aerial Platform (MAP)

The MAP is converted from a manned helicopter or fixed-wing aircraft, by mounting to it the phenotype acquisition kit, placed in a cargo pod or directly installed on the step (via a bracket), with a passenger(s) evaluating the images and giving feedback on their quality to the pilot, in real time, *via* a video monitor in the cockpit. The phenotypic equipment used for this entail sensors, GPS unit, gyroscopes and/or inertial measurement units. Although the MAP is capable of carrying multiple sensors for complicated phenotypic tasks, this has not been fully exploited, likely because it would depend on much manual participation. For example, just one radiometric calibration thermal camera in the cargo pod of a helicopter was employed for collecting canopy temperature (Deery et al., 2016)—and a passenger must be present to perform the image assessment and provide feedback. For this, an RGB camera can be remotely controlled and triggered by an operator, to watch the “real situation” through a video monitor in the cockpit (Yang and Hoffmann, 2015). For estimating the aboveground biomass (AGB) and underground biomass (BGB) of maize, LiDAR was also installed on a MAP, to collect point-cloud data at the nominal height of 1,500 m (Li et al., 2015). High imaging heights and airspeeds certainly make the acquisition of phenotypic data at a high resolution and accuracy more challenging.

Similarly, MAP can also make effective use of pre-existing agricultural machinery, and can overcome challenging weather conditions to a certain extent because of flight stability. Nevertheless, conducting phenotypic experiments with MAP demands a specific amount of manpower and inevitably involves high costs. For example, MAP requires a trained pilot with relevant qualifications to operate the helicopter or plane, and a passenger on board doing the monitoring, assessing, communicating, and manual imaging. Moreover, considering the cost and technical issues, the advantages of MAP have not been fully exploited (i.e., flight altitude, flight speed, carrying capacity). Perhaps that's why MAP's prominence has dropped sharply in crop phenotyping, precisely because of UAP's unparalleled advantages, which enable it to complete the consistent phenotypic tasks and thus progressively supplant MAP.

#### Satellite Platform

Satellites can provide panchromatic imagery, multispectral imagery, or radio detection and ranging (RADAR) data. Panchromatic images of a single-band are displayed as gray-scale images with high resolution but limited spectral information, whereas multispectral images have a rich spectrum yet with relatively low resolution. Thus, these obtained panchromatic and multispectral images are usually merged by panchromatic sharpening or pan-sharpening, to obtain multispectral raster data having a high resolution. However, such optical satellite phenotyping is susceptible to uncooperative weather conditions, such as cloudy, rain, fog, and haze, and it also suffers from visible light saturation (Jin et al., 2015). In this case, RADAR and synthetic aperture radar (SAR) data are able compensate for this defect extremely well. This is due to the unique sensitivity of crop structure to microwaves, which effectively improves the availability of the satellite platform. WorldView series, RapidEye, GeoEye-1, SPOT series, QuickBird, Ikonos, Planet Scope, Pleiades series, KOMPSAT series, Satellite for Earth Observation series, Landsat series, Gaofen series, and SkySat series are currently the main satellite HT3Ps that can obtain color and multispectral images (Jin et al., 2020). Those satellite platforms able to obtain RADAR data are mainly Sentinel-1, RADARSAT-2, ENVISAT, TerraSAR-X/TanDEM-X, and RISAT-2 (Zhang et al., 2020).

In recent years, with the rapid development of satellite HT3Ps, there is increasingly more research on plant phenotyping done using satellite data. For example, multispectral data of GeoEye-1 satellite was used to evaluate the nitrogen status and spatial variability of different species of turfgrass (Caturegli et al., 2015), leading to an important guiding principle for turf fertilization management. The RapidEye satellite provides five bands with a spatial resolution of 5 m: blue, green, red, red-edge, and near infrared. Its multispectral images were used to study the variable rate of wheat nitrogen fertilizer effects (Basso et al., 2016). In evaluating the crop angle of indentation (CAI), Chauhan et al. (2020) relied on Sentinel-1 and radarsat-2 (multi-incidence angle) data and went on to evaluate the severity of crop lodging. An interesting satellite platform, FLuorescence EXplorer (FLEX), is equipped with a single payload fluorescence imaging spectrometer (FLORIS) that combines spectral and spatial resolution to retrieve and interpret the full chlorophyll fluorescence spectra emitted by terrestrial vegetation (Drusch et al., 2017). It is scheduled to launch in 2022 and will fly in the same orbit as Sentinel-3; hence, the availability and interoperability of auxiliary information.

The ability to process large-scale satellite phenotypic data at low cost according to international standard protocols is a key advantage of satellite phenotyping. Some satellites provide free data to would-be users, but the acquisition of high-precision commercial satellite data normally has a monetary charge. Fortunately, the cost of accessing to satellite data is now modest. Still, both panchromatic and multispectral imaging done by satellites remains susceptible to interference of atmospheric, clouds, and fog. In addition, the resolution issue—such as Pleiades-1a, 0.5 m; SPOT 6, 1.5 m; Planet Scope 3.0 m; Rapid Eye, 5.0 m, to name a few—and the data revisit period are also limiting factors. Fortunately, satellite resolution is making continuous progress as satellite technology advances. For instance, the Finnish technology start-up ICEYE has released commercial spaceborne SAR samples with the highest resolution (0.25 m) currently available worldwide. In the near future, low-orbiting nanosatellites and microsatellites with high spatiotemporal resolution may become join the prevailing aeronautical HT3Ps.

## HT3PS' Combination for Comparative Validation or Comprehensive Analysis

**Figure 4:**
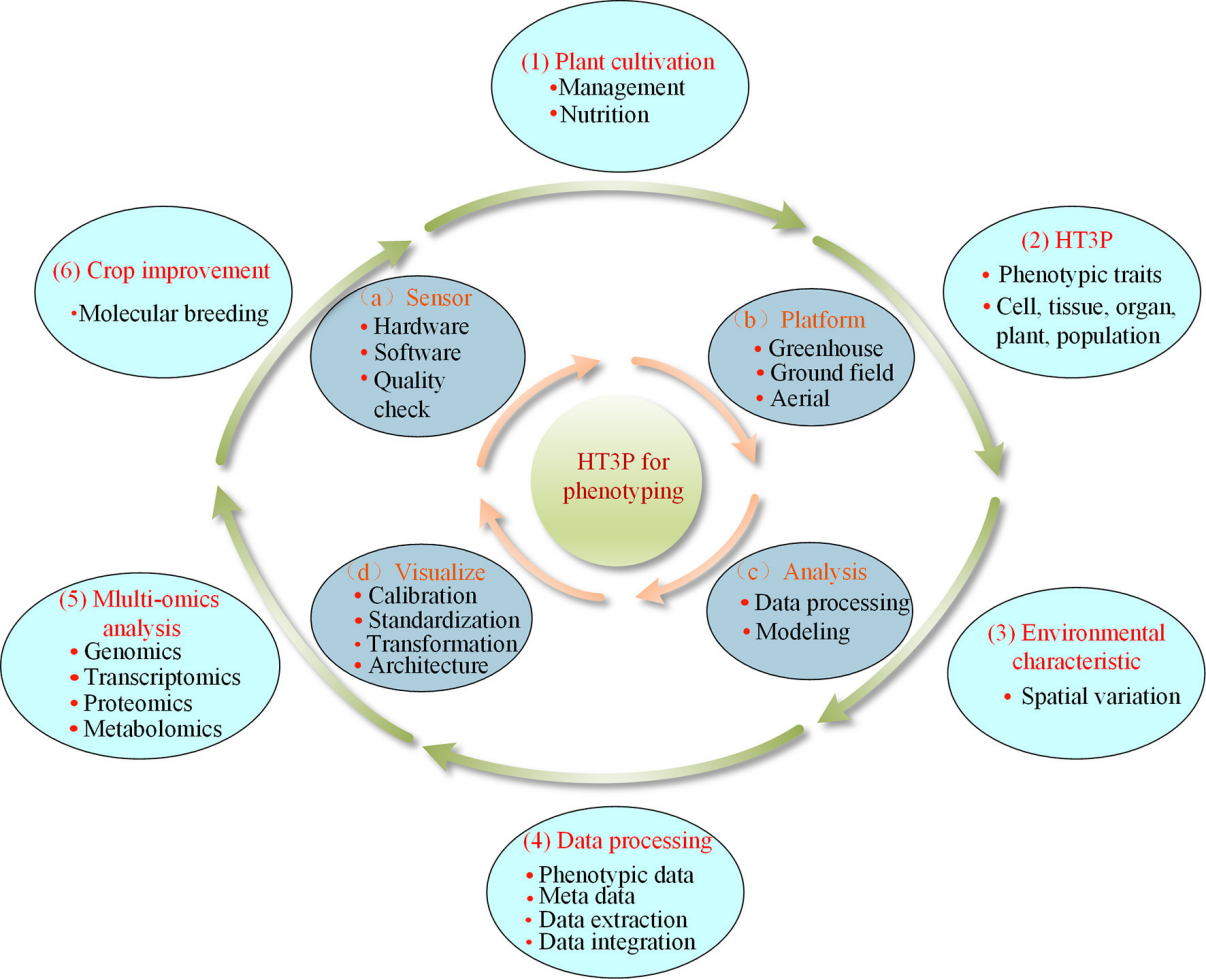
Typical comprehensive HT3P components: (a) sensor, (b) platform, (c) analysis, (d) visualization; HT3P performs the entire workflow of phenotyping: (1) cultivation of plants, (2) extraction of phenotypic traits, (3) acquisition of environmental parameters, (4) data processing, (5) multi-omics analysis, and, ultimately, (6) aiding in crop improvement.

A typical comprehensive HT3P consists of four components: sensor, platform, analysis, and visualization. Together, these should be able to perform high-throughput and non-destructive acquisition of a substantial amount of data on dynamic phenotypic traits of cultivated plants and their environmental characteristics, as well as providing multi-omics analyses, completing the entire phenotyping process from a holistic perspective, thereby ultimately facilitating crop improvement and molecular breeding of plants (as shown in Figure 4). Since the various types of HT3Ps each have their own unique merits—Table 4 summaries the advantages and disadvantages of miscellaneous HT3Ps—with traditional manual measurements usually needed to take relative real data for comparison and verification, pursuing combinations of differing HT3Ps offers a way to break away from traditional phenotyping and obtain comprehensive high-precision phenotypic data. Several common and typical combinations of diverse HT3Ps are highlighted in Table 5.

**Table 4:** Overview of the comparison of advantages and disadvantages of the different types of HT3Ps.

**HT3P type**	**Advantages**	**Disadvantages**
Benchtop-type	Strong repeatability; continuous monitoring; precision phenotyping; high resolution	Expensive; for small plants only
Conveyor-type	Large sample size; strong repeatability; high resolution	Expensive; high operating costs
Pole/tower-based	Low cost; relatively simple structure; flexible movement	Small range; increased distance decreases resolution
Mobile	Semi-automatic or fully automatic; high resolution; expansive; high flexibility.	Affected by weather, soil conditions; soil compaction; mechanical interference; requires some manpower; long boom may cause sensor jittering and blurred images; safety mechanism needed
Gantry-based	Low weather dependency; continuous phenotyping all-day	Expensive; fixed limited area; high maintenance costs
Cable-suspended	Low weather dependency	Expensive; fixed limited area; limited endurance
UAP	Flexible flight plan; coverage a wide range of field plots; relatively low cost; GPS navigation;	Weather (light, rain, fog, etc.) dependence; limited payload; limited endurance; strict aviation regulation (altitude); flight training
MAP	Flexible payload; rapid coverage of large areas	Expensive; non-repeatable flight route; substantial manpower
Satellite	Coverage a wide range of field plots; relatively low cost	Low resolution; long return period; weather restrictions (except radar)

**Table 5:** Overview of typical applications of HT3Ps when used in combination.

**Combination type (a+b)**	**Sensors^**a**^**	**Sensors^**b**^**	**Plants**	**Traits**	**References**
Mobile + pole/tower-based	Stereo camera	RGB, infrared	Maize, sorghum	Plant height, leaf area index (LAI)	Shafiekhani et al., 2017
UAP + mobile	RGB	LiDAR	Wheat	Plant height	Madec et al., 2017
MAP + mobile	Monochromatic, thermal	Sonar, IR radiometer, multispectral	Cotton	Canopy height, canopy temperature	Andrade-Sanchez et al., 2014
MAP + pole/tower-based	Thermal IR	IR thermometer	Wheat	Canopy temperature	Deery et al., 2019
UAP + UAP	RGB	Hyperspectral	Crop	–	Habib et al., 2017
UAP + satellite	RGB, thermal, multispectral	Multispectral (VNIR, SWIR)	Soybean	Mean canopy temperature, water stress resistance, VIs	Sagan et al., 2019b

### HT3Ps' Combination for Comparative Validation

Some combinations of HT3Ps seek to compare and validate the phenotypic performance by applying different types of HT3Ps and thereby bypass traditional manual measurements entirely. For instance, Andrade-Sanchez et al. (2014) modified a sprayer to function as a mobile platform, to simultaneously collect canopy height, reflectance, and temperature in four adjacent rows in a cotton field; it can carry four sets of sensors, including sonar sensors, IR radiometers, and multispectral canopy sensors. Then, to verify the data authenticity of the mobile platform, a MAP (helicopter) was applied to collect visible-near-infrared (VNIR) and thermal IR data. In other work, to evaluate the plant height of wheat, a mobile platform with LiDAR and a UAP with RGB cameras were jointly used to generate 3D dense-point clouds (Madec et al., 2017). However, because of the low resolution of RGB images from UAP and the strong penetrability of LiDAR of the mobile platform, plant height measurement based on UAP was underestimated. Similarly, Khan et al. (2018) combined a mobile platform and a UAP to collect RGB images, which revealed that canopy height obtained from the mobile platform is more accurate than UAP, whereas the plant vigor evaluated from the UAP is more precise. A multi-rotor UAP, with an RGB camera and a fixed-wing UAP with a hyperspectral push-broom scanner, was devised by Habib et al. (2017) to verify the feasibility of using RGB-based orthophotos to improve the geometric features of hyperspectral orthophotos. In addition, the combination of a UAP and four satellites was implemented to compare the phenotypic capabilities of different resolutions in dry bean (Sankaran et al., 2019), whose results indicated that using sub-meter resolution satellites as HT3Ps holds promising application prospects for field crop phenotyping.

While some combinations of different types HT3Ps are still based on the time-consuming and laborious traditional field measurements, these will gradually disappear with the stabilization and improvement of the advanced HT3Ps. The combination of various types of HT3Ps for cross-validation is gradually moving forward, representing a landmark step in the field of plant phenotyping.

### HT3Ps' Combination for Comprehensive Analysis

Some HT3Ps' performance aspects are combined to realize the fusion of multi-source data for collaborative and comprehensive phenotyping. For example, using both a mobile platform and a tower-based platform for canopy scale and single plant phenotyping has been proposed by Shafiekhani et al. (2017). As an autonomous mobile platform, Vinobot, with its stereo cameras installed on the robotic arm, can autonomously navigate in the field and collect data on individual plant traits. The tower-based Vinoculer can inspect a large-area canopy phenotype, and delegate specific regions to Vinobot for elaborate phenotyping, which greatly improves the flexibility and purposiveness. A robotic mobile platform and a UAP collected complementary multispectral data, which let investigators obtain comprehensive crop phenotypes (Ingunn et al., 2017). Specifically, that mobile platform provided detailed traits information of plants and the UAP obtained calibrated NDVI, and together they further predicted the heading date and yield. One multi-rotor UAP with an RGB camera, and another with a multispectral camera, were combined to monitor tomato crops, so as to formulate management measures and determine the best management scheme for specific fields (Marconi et al., 2019). Likewise, Anderson et al. (2020) employed a rotary-wing UAP and a fixed-wing UAP to monitor a track of plant height growth of an recombinant inbred line (RIL) maize population; hence, they could, for the first time, elucidate dynamic characteristics of quantitative trait loci (QTL) in real time under the field conditions. In addition, Deery et al. (2019) designed a MAP with airborne thermal IR cameras and a pole-based platform (Arducrop wireless IR thermometers) and used it to continuously measure crop CT (canopy temperature). To fill the temporal gap in the availability of satellite data and improve the usability of UAP, Sagan et al. (2019b) merged RGB, thermal, and multi-spectral images from UAP with VNIR and SWIR imagery taken by the WorldView-3 satellite, applying them to crop monitoring and early stress detection on a temporal scale, which contributed to form field-scale coordinated data of UAV and satellite virtual constellation.

The imaging distance of various HT3Ps will engender a differing spatial resolution and pixel size. Taking ground coverage (GC) as an example, the pixel size of an RGB image has a huge impact on the accuracy of GC's evaluation (Hu et al., 2019). In addition, there are significant disparities among different types of HT3Ps, such as their time resolution, weather dependence, experimental scale, and financial investment, to name a few. This means that combinations of HT3Ps ought to steer toward actual phenotypic requirements and concrete practical issues. Along with further refinement of plant phenotyping, the future HT3P portfolio is expected to integrate multi-site distributed platforms, single-point centralized platforms, and cloud-based platforms, to deeply mine and dissect multi-source phenotypic data from dynamic time series at multiple scales, for multiple species, and under multiple scenarios. Furthermore, to effectively integrate existing HT3Ps, phenotyping technology, phenotypic methods, data availability, and resources, to speed up the emergence of high-quality phenotypic achievements and accelerate crop breeding, while also reducing duplication of research and investment, more international and regional organizations, or initiatives (see Table 6) have come into being.

**Table 6:** Overview of international organizations or regional initiatives contributing to plant phenotyping.

**Organization acronym**	**Full name (or description)**	**URL**
APPF	Australia Plant Phenomics Facility	https://www.plantphenomics.org.au/
APPN	Austrian Plant Phenotyping Network	https://appn.at/
CGIAR	Modernize breeding programs targeting the developing world	http://excellenceinbreeding.org/
CIMMYT	International Wheat and Maize Improvement Center	https://www.cimmyt.org/
CPPN	China Plant Phenotyping Network	–
CSISA	The Cereal Systems Initiative for South Asia	https://csisa.org/
DPPN	German Plant Phenotyping Network	https://dppn.plant-phenotyping-network.de/
EMPHASIS	European Plant Phenotyping Infrastructure	https://emphasis.plant-phenotyping.eu/
EPPN2020	European Plant Phenotyping Network (2020)	https://eppn2020.plant-phenotyping.eu
ESFRI	European Strategy Forum for Research Infrastructure	https://www.esfri.eu/
FPPN/PHENOME	French Plant Phenomic Network	https://www6.dijon.inrae.fr/umragroecologie_eng/Research-Programs/Investissement-Avenir/PHENOME
LatPPN	Latin American Plant Phenomics Network	–
LEPSE	Laboratory of Plant Ecophysiological Responses to Environmental Stresses	http://www1.montpellier.inra.fr/ibip/lepse/english/
NAPPN	The North American Plant Phenotyping Network	http://nappn.plant-phenotyping.org/
NPPN	Nordic Plant Phenotyping Network	https://nordicphenotyping.org/
NPEC	Netherlands Plant Eco-phenotyping Centre	https://www.wur.nl/en/product/TheNetherlands-Plant-Eco-phenotypingCentre-NPEC.htm
G2F	The Genomes to Fields Initiative	https://www.genomes2fields.org/
GCN	Green Crop Network	http://www.greencropnetwork.com/
IPPN	International Plant Phenotyping Network	https://www.plant-phenotyping.org/
JPPC	The Jülich Plant Phenotyping Centre	http://www.fz-juelich.de/ibg/ibg-2/EN/Research/Phenotyping/Phenotyping_article.html?nn=548814
MIAPPE	Minimum Information About a Plant Phenotyping Experiment	https://www.miappe.org/
PHEN-ITALY	Italian Plant Phenotyping Network	http://www.phen-italy.it/index.php
PhenomUK	Promotes an integrated, holistic view of the phenotyping process across the UK	https://www.phenomuk.net/
TERRAREF	Terraphenotyping Reference Platform	https://www.terraref.org/
Wheat Initiative	Endorsed by the G20 Agricultural Ministers, to contribute to improving world food security	https://www.wheatinitiative.org/our-vision

## Simulation HT3P

The simulated HT3P aims to model plant growth, phenotypic expression, and phenotyping at various scales. It does this by integrating multi-source information in a modeling framework, such as that of germplasm resources, irrigation, fertilization, nutritional substance, spatial climate, soil environment, terrain properties and management records. For example, a digital plant phenotyping platform (D3P) would use environmental variables, crop management, and meteorological information as input, to generate 3D virtual canopy structure. Based on this, the collection of virtual canopy phenotypic traits can be performed by RGB, multi-spectral, and LiDAR simulators (Liu et al., 2019). For whole forests with large cover areas, long-lived cycles and high heterogeneity, Dungey et al. (2018) provided a prototype of a landscape-scale HT3P simulator, by consolidating remote sensing topography, environmental impacts, spatial abiotic information, management records and genomics into the modeling framework. It aimed to eliminate some traditional limitations in tree breeding programs and provide genetic gains in tree fitness.

By combining genomics, high-throughput phenotyping, and simulation modeling, we can obtain an adequate but sound understanding of phenotypic traits and their variation (Varshney et al., 2018). The application of various complex models to combine simulations with empirical methods will contribute markedly to accelerating the process of extracting ideal phenotypic traits for use in crop improvement.

## Future Prospects for HT3P

The concept of HT3P is rather grand, such that the development and innovation of HT3Ps depends on the cross-disciplinary cooperation of agronomy, robotics, computer, automation, artificial intelligence, and big data, requiring the participation of experts—breeders, agronomists, plant scientists, mechanical engineers—and leadership from interdisciplinary talent of open innovation teams. Whether HT3P is phenotyping in the greenhouse or in the field, ground-based proximal phenotyping or aerial large-scale remote sensing, the future of HT3Ps lies in improving spatial-temporal resolution, sensor integration, turnaround time in data analysis, human-machine interaction, operational stability, throughput, automation, operability, and accessibility.

It is worth noting that the development, selection, and utilization of HT3Ps should be orientated by concrete project requirements, specific phenotypic tasks, and practical application scenarios, such as the field coverage (Kim, 2020), rather than assuming that more devices, technologies, and funds with which the HT3P is equipped, the better; partly because the collection of a large amount of data does not mean all of it is useful (Haagsma et al., 2020). Even in some cases, the experimental effects of applying single and multiple sensors are identical (Meacham-Hensold et al., 2020), and the data obtained from multiple devices are redundant. However, the combinations of various HT3Ps for comparative validation and comprehensive analysis could provide broad application prospects for inspection, extraction, and quantification of complex physiological functional phenotypes. Yet the technical issues of formulating standards and synchronizing calibrations for these multiple combinations are daunting tasks. Fortunately, the involvement of meta-analysis ensures the objectivity of HT3P development and selection. For example, Young (2019) applied meta-analysis method to develop an evaluation framework that can quantitatively and objectively assess the complexity and utility scores of high-throughput systems. As an effective analytical method of quantitative, scientific synthesis of research results (Gurevitch et al., 2018), meta-analysis may prove especially fruitful in the near future.

Specifically, the future conveyor-type HT3P requires consideration of operational stability and environmental homogeneity, and allowing phenotypic analysis for multi-level subtle traits of a wide variety of representative plants will be a key design factor to the development of the future benchtop-type HT3P. Scalability, rotatability and multi-site deployment will be the prospective features of pole/tower-based HT3Ps, and economically-efficient distributed ones will perform outstandingly in the calibration of high-dimensional phenotypic data. Mobile HT3Ps that can be transported to the experimental site are preferred by phenotypic researchers rather than the experiment coming to the limited platform (Roitsch et al., 2019). This means that the development of mobile HT3P needs to move toward flexibility and portability, and that modular and customizable design will be welcomed by the phenotyping community. The reduction of volume and cost is the major consideration for future gantry-based HT3P designs, and the new cable-suspended HT3P will have the ability to monitor continuously and consistently crop growth and development at low altitudes over long periods of time. As for UAP, the development of compact lightweight sensor configuration that is sensitive to plant-specific phenotypic traits will be a breakout (Xie and Yang, 2020). In addition, advanced battery technology is in dire need of a stage breakthrough, which can greatly improve the endurance, payload, and power of the UAV. For satellite phenotyping, improving image resolution and shortening the revisit cycle remain the focus of satellite platform development. Additionally, cost-effective platforms also warrant consideration, as smart phone, handheld portable instrument, backpack system, and wearable device are adopted and updated for utilization in phenotyping.

High-throughput data acquisition, data management, data interpretation, modeling, integration, and application together form the core and pillar of plant phenotyping. The main challenges faced by the new generation of phenotyping are data handling, images processing, and traits analyzing (Fahlgren et al., 2015a; Campbell et al., 2018; Hickey et al., 2019). Fortunately, the introduction of various software, web-based tools, pipelines, toolkits, deep learning tools, and online repository solutions to assist phenotypic researchers in processing phenotypic data will break through these technical bottlenecks. For example, a free multi-purpose software—Coverage Tool—can semi-automatically quantify a wide range of visual plant traits (Merchuk-Ovnat et al., 2019). Web-based image analysis tools, such as Field Phenomics (Guzman et al., 2015), are considered by us to be a hotspot for phenotypic solutions. Kar et al. (2020) developed an analysis pipeline with outlier detection, missing value imputation, and spatial adjustment for solving the problem of inaccurate and missing phenotypic data. Toolkits tend to be relatively specific, such as Plant 3D (P3D), which specializes in analyzing 3D point cloud data of plant structures (Ziamtsov and Navlakha, 2020). With the advancement of HT3P, their improving high-throughput and efficiency will produce increasingly big data. For huge datasets, deep learning tools are needed; however, only when large datasets that capture shared problems become available can the greatest benefit be gained from the application of deep learning tools. Online databases, such as http://www.plant-image-analysis.org, can effectively bridge the gap between developers and users, but still lack comprehensive management platforms that cover software, web-based tools, pipelines, toolkits, deep learning tools, and other phenotypic solutions, which will be a milestone breakthrough as well as a considerable challenge.

With the emergence of various HT3Ps, experimental designs, phenotyping methods, standardized management, both phenotype acquisition and its data analysis are becoming extremely prominent. Phenotypic data that costs substantial capital, labor, time, and energy, however, may 1 day be abandoned forever (Mir et al., 2019). Presently, a standard phenotyping agreement or data analysis methodology for plant phenotyping has yet to be established (Mahlein et al., 2019). The standardization of data and metadata from the HT3Ps contributes to an improved data utilization rate and it ensures the interoperability of data providers and experimental replication. Otherwise, data that is poor annotated and in a disorderly format may generate noise or disordered waves. Fortunately, relevant standard constraints are being proposed. For example, Krajewski et al. (2015) published a technical paper offering effective recommendations (at http://cropnet.pl/phenotypes) and initiatives (such as http://wheatis.org), making a further step toward establishing internationally practical solutions. Moreover, originating the relevant standardization of phenotyping can strengthen the comprehension and explanation of biological phenomena, contributing to the transformation of biological knowledge and establishment of a real coherent semantic network.

## Conclusions

HT3P is a novel and powerful tool for obtaining plant-deep phenotypes (morphological structure, physiological function, component content) and dense phenotypes in complex field setting, which cannot be accomplished by traditional phenotyping approaches. This paper reviewed the application of HT3Ps in the growth chamber or greenhouse with strictly controlled environmental conditions and field phenotyping with notoriously heterogeneous conditions and uncontrollable environmental factors. Then, according to platform configuration and operation mode, further classifications were performed to provide comprehensive overview and description and assessment of the various types of HT3Ps currently available. The unique characteristics, applications, and strengths and weaknesses of various HT3Ps were emphasized. Going further, the simulation platform, various combinations of HT3Ps for comparative validation or comprehensive analysis, current phenotypic challenges, and the future development trends of HT3Ps were discussed.

With the assistance of powerful HT3Ps, phenomics has arguably entered a new stage (Tardieu et al., 2017). At this stage, the new and pressing challenge of next generation phenotyping will be to reasonably combine phenotypic experiments, various HT3Ps, models, data processing and handling scheme, meta-analysis, and visualization of phenotypic information for optimizing the allocation of research resources, efficiently accomplishing complex phenotypic tasks, and transforming massive multi-source phenotypic data into statistical and biological knowledge. Robust phenotyping is central to plant breeding (Hickey et al., 2019), and the development of satisfying crop varieties with high-yielding and strong stress resistance is the ultimate goal of crop breeding. High-throughput sequencing activity underpins the fast development of genomics (Shah et al., 2018). Likewise, HT3P as a novel and powerful phenotyping tool will explore a new period of rapid development in phenomics. Further, combining morphological, physiological, and elemental phenotyping with multi-omics methods from the perspective of holistic omics will usher in a new era of botany phenotyping.

## Author Contributions

DL and CL guided the writing of the article and reviewed the initial versions of it. CQ completed the manuscript's writing content and graphic production. ZS and GY gave comments on the framework and diagrams of the article. XL was involved in both language correction and graphic modification of the article. AM assisted in improving word accuracy and language expressiveness. All authors have made substantial contributions to the conception, drafting of the manuscript, read, and approved the submitted version of the article.

## Conflict of Interest

The authors declare that the research was conducted in the absence of any commercial or financial relationships that could be construed as a potential conflict of interest.
